# Exploring the impacts of implicit context association and arithmetic booster in impulsivity reduction

**DOI:** 10.3389/fpsyt.2022.961484

**Published:** 2022-09-13

**Authors:** Minho Hwang, Sung-Phil Kim, Dongil Chung

**Affiliations:** Department of Biomedical Engineering, Ulsan National Institute of Science and Technology (UNIST), Ulsan, South Korea

**Keywords:** impulsivity, associative memory, working memory, cognitive training, task repetition effect

## Abstract

People have a higher preference for immediate over delayed rewards, and it is suggested that such an impulsive tendency is governed by one’s ability to simulate future rewards. Consistent with this view, recent studies have shown that enforcing individuals to focus on episodic future thoughts reduces their impulsivity. Inspired by these reports, we hypothesized that administration of a simple cognitive task linked to future thinking might effectively modulate individuals’ delay discounting. Specifically, we used one associative memory task targeting intervention of context information, and one working memory task targeting enhancement of individual’s ability to construct a coherent future event. To measure whether each type of cognitive task reduces individuals’ impulsivity, a classic intertemporal choice task was used to quantify individuals’ baseline and post-intervention impulsivity. Across two experiments and data from 216 healthy young adult participants, we observed that the impacts of intervention tasks were inconsistent. Still, we observed a significant task repetition effect such that the participants showed more patient choices in the second impulsivity assessment. In conclusion, there was no clear evidence supporting that our suggested intervention tasks reduce individuals’ impulsivity, and that the current results call attention to the importance of taking into account task repetition effects in studying the impacts of cognitive training and intervention.

## Introduction

In daily decision-making, people sometimes make a choice not because it is better now but because it will bring them a higher return in the future. One’s exaggerated preference for temporally proximate small reward over delayed large reward has been referred to as impulsivity (1–4), and choices that show the opposite pattern, decisions with consideration of future consequences, are considered as outcomes of successful self-control (5–7). A large body of studies examined the tradeoffs between time of reward delivery and amount of reward, and suggested that discounting the value of delayed rewards explains why typical individuals often show seemingly impulsive behaviors, i.e., choosing the small immediate reward over the large delayed reward (8–10). This framework of temporal discounting in valuation extends to real-life health-risk behaviors such as substance abuse (2, 3, 11, 12), problem gambling (13, 14), and internet addiction (15). Consistent with the view that one discounts delayed rewards, individuals who tend to make choices that may deliver immediate pleasure than long-term healthiness (e.g., eating a chocolate bar instead of an apple as dessert) also showed higher discounting rates in decision-making tasks (16, 17).

A classic intertemporal choice (ITC) task has been used to capture individuals’ behavioral traits across a broad range of mental illnesses (18). Notably, behavioral traits characterized by the task (i.e., delay discounting) were able to dissociate whether or not individuals with psychiatric illness have comorbid substance use problems (19–21). Building based on this association, previous studies have suggested some methods that could potentially be used as systematic intervention designs for individuals who show problem behaviors (e.g., substance use). Specifically, recent studies found evidence suggesting that one’s ability to simulate future rewards is linked with the extent to which one is sensitive to temporal delay of rewards (22–26). Individuals’ preference for delayed large rewards significantly increased when they were explicitly instructed to imagine the reception of the reward in association with a cue selected from their episodic future plans (e.g., trip to Paris). These findings supported that episodic future thinking induces vivid imagination (simulation) of future reward, and, in turn, reduces reward delay discounting.

During the recent outbreak of the COVID-19 pandemic, individuals’ smoking and alcohol consumption amount increased (27). As active mitigation of such health risk behaviors, various attempts have been made to develop digital solutions (28–30). Based on previous laboratory-based studies, episodic future thinking is expected to reduce individuals’ impulsive choice tendency, a typical hallmark of individuals who suffer from substance use disorder (2, 3, 11, 12) or problem gambling (13, 14) and considered as a feasible digital solution for individuals with addiction. However, recent reports were inconsistent such that not all studies were able to find significant effects of the intervention (31, 32). Such mixed results might be stemming from the complex nature of cognitive processes involved during the episodic future thinking intervention and from our lack of understanding of the mechanisms by which the induction successfully reduces individuals’ delay discounting (33). Here, we suggest two simple cognitive properties that are closely related to mental simulation and examine the effects of mediating them on reducing individuals’ impulsivity. Given the perspective that future reward simulation requires cognitive construction of hypothetical scenarios or reconstruction of real events (26, 34), we set (i) increasing the amount of context information and (ii) enhancing individuals’ working memory capacity as two potential intervention targets.

Our first target for intervention design is the amount of information regarding the simulated context. A recent study showed that episodic future thinking has a different impact on delay discounting depending on its content (35). Other studies suggested that reduction in future uncertainty may account for the mechanism of episodic future thinking (36), and that individuals’ tendency to avoid uncertainty is associated with the extent to which they discount delayed rewards (37). Based on these previous studies, we speculate that the core mechanism by which episodic future thinking reduces impulsivity (24) might be the facilitation of delayed reward simulation by linking information from individuals’ own past experiences to a delayed time point (38). If so, one might expect a comparable simulation effect from additional information that has a context helpful for mental simulation but not necessarily linked to personal experience. Specifically, we hypothesized that associating delays for potential rewards with additional context information (travel locations) may facilitate future reward simulation without being instructed explicitly to participate in episodic future thinking and, in turn, reduce individuals’ impulsivity.

Working memory capacity, our second target for intervention, is known to play a pivotal role in mental simulation. Previous studies have suggested that increased working memory capacity may facilitate the simulation process (39, 40). Furthermore, in a recent study, Snider et al. showed that when combined with working memory training, the impact of episodic future thinking on reducing individuals’ impulsivity was elevated (41). Data from a large sample indicated that there is no significant direct relationship between individuals’ baseline working memory capacity and their impulsivity (42). However, changes in individuals’ capacity had a significant impact on their impulsivity. Bickel et al. (43) introduced multiple sessions of working memory training to participants and showed that the delay discounting tendency of individuals who have stimulant use disorder can significantly be reduced. In line with the study, working memory training in adolescents with low socioeconomic status lowered their delay discounting tendency (44). These results suggest that working memory training may be a potential intervention to reduce individuals’ impulsivity.

**FIGURE 1:**
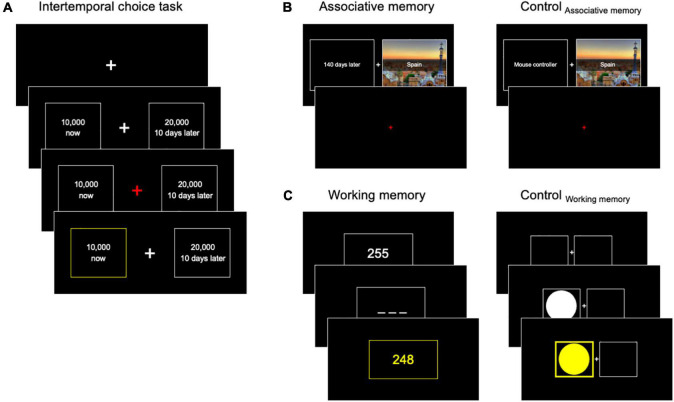
Intertemporal choice task and cognitive intervention paradigms. **(A)** During the intertemporal choice (ITC) task, the participants made choices between an immediate smaller reward and a delayed larger reward. The immediate reward was fixed (10,000 Korean Won, KRW), but the alternative delayed option was constructed using six distinct delays (1, 10, 21, 50, 90, and 180 days) and varying sizes of delayed rewards (10, 300–29, 300 KRW) determined by stepwise approach. Individuals could make a response when the crosshair turned red, and a feedback screen followed their choices to highlight the chosen option. **(B,C)** Two intervention tasks and their control tasks were used to examine the impacts of associative memory and working memory on future reward simulation and, in turn, on delay discounting. (**B**, left) The associative memory task comprised the encoding and retrieval phases (not depicted here). In the encoding phase, the participants were asked to memorize associations between the paired stimuli. Specifically, names of countries with a picture of their representative must-see sights were matched with “expected preparation time” to go on a trip to the places. Note that the preparation time was presented in the same format as the reward delays used in the ITC task (e.g., 10 days later). (**B**, right) The Control_AM_ was largely identical to the Associative memory task, except that the associations were made between country names and various stationaries. (**C**, left) In the working memory task, the participants had to remember a random three-digit number presented on the screen (e.g., 255), mentally subtract seven, and report the number on the subsequent screen (e.g., 248). They were asked to repeat this mental calculation (e.g., 241, 234, 237, …) until a new random number was provided. (**C**, right) During the Control_WM_ task, the participants had to maintain a sustained level of attention. Specifically, in each trial, a circle showed up on either left or right half of the screen, and the participants were asked to press the arrow key that matches the location as soon as possible.

These hypotheses were tested across two independent experiments by examining whether modulation of each factor indeed significantly reduced individuals’ delay discounting, which is an indication of successful choice impulsivity reduction. We first administered a classic ITC task [adapted from Kable and Glimcher (4)] (Figure 1A and Supplementary Figure 1a) and measured individuals’ baseline delay discounting. During the ITC task, the participants made a series of choices between a fixed immediate reward of 10,000 KRW (around $10) and a larger delayed reward that varied from trial to trial (refer to section “Experimental procedures” for detailed experimental procedures). Previous studies have suggested that an individual’s choice between delayed and immediate reward options can be accounted for by comparison of the options’ subjective values (SVs) defined as hyperbolic discount function SV = V/[1 + *k*D] (8, 9, 45), where V indicates an objective reward, D indicates the delay, and *k* is each individual’s delay discount rate. To quantify individuals’ impulsivity, we estimated their delay discount rates based on participants’ choices across three sessions of the ITC task. In addition to measuring the impacts of modulating each factor on impulsivity reduction, we examined individuals’ impulsivity changes across repeated task-based measures to evaluate the potential impacts of choice monitoring.

To examine whether individuals’ impulsivity can be modulated, we introduced one of five types of intervention tasks (associative memory task, working memory task, and three control tasks) after the baseline ITC task and then a second assessment of ITC task (Experiment 1). Furthermore, to examine whether the results from experiment 1 are replicable, we conducted another independent experiment where we tested three specific types of intervention tasks among the original five (Experiment 2).

## General methods

### Participants

A total of 216 healthy young adults (male/female = 124/92) participated in the current study. None of the participants reported a history of neurological or psychiatric illness. Two independent studies were conducted, and there were no overlapping participants across the two experiments. A total of 126 healthy individuals (male/female = 71/55, age = 22 ± 2.48) participated in experiment 1 conducted between 25 March and 20 May 2019, and 90 individuals (male/female = 53/37, age = 22.24 ± 3.93) were recruited for experiment 2, conducted between 13 January and 19 July 2021. All the participants provided written informed consent and were paid for their participation. The study was approved by the Institutional Review Boards of Ulsan National Institute of Science and Technology (UNISTIRB-18-18-A). No statistical methods were used to predetermine sample sizes, but our N for each intervention task group was similar to those reported in previous intervention studies (24, 41, 43, 46, 47).

### Experimental procedures

The aim of the current study was to measure individuals’ impulsivity and examine whether the intervention of cognitive factors alters their characteristics. In the beginning of each individual’s participation, he or she was randomly assigned to one of the five subgroups (three subgroups for Experiment 2); each of which was paired with one type of intervention task. All the participants completed one session of the intertemporal choice task (ITC), one session of the assigned intervention task, and one additional session of ITC. The participants were paid at the end of the study based on the delay and reward of a single random choice drawn from all the ITC task decisions the participants made. All the experiments were performed in accordance with relevant guidelines and regulations.

#### Intertemporal choice task

During the ITC task, the participants made a series of choices between a fixed immediate reward of 10,000 KRW (around $10) and a larger delayed reward that varied from trial to trial (Figure 1A and Supplementary Figure 1a). Six distinct delays (1, 10, 21, 50, 90, and 180 days) were used for the delayed reward option, and the value of the option was determined following the staircase approach. There were two unique initial values for the large delayed reward (15,000 and 20,000 KRW), and the staircase approach was used for each pair of [delay, initial value]. Specifically, if a participant chose the immediate option at the n^th^ step, the reward of the delayed option was set to [delayed reward at the n^th^ step ^+^ 10,000 × (1/2)^n–1^] (n = 1, 2, 3, 4, 5) for the next trial where the same pair of [delay, initial value] was used. On the contrary, if a participant chose the delayed option, the reward of the delayed option was set to [delayed reward at the n*^th^* step ^–^ 10,000 × (1/2)^n–1^] for the next trial where the same pair of [delay, initial value] was used. There was one exception. To avoid the delayed reward being equal to or smaller than the immediate reward (10,000 KRW), choices of the delayed option on the trials under the initial value of 15,000 KRW led to the next trial with the delayed reward of [delayed reward at the n^th^ step – (delayed reward at the n^th^ step – 10,000) × (1/2)^n–1^]. This “titration” procedure is repeated in five iterations (steps) for each distinct pair of delay and initial value. Each block comprised 60 trials ([6 delays] × [2 unique initial values for the large reward (15,000 and 20,000 KRW)] × [5 iteration staircase]). To minimize the effect of participants remembering previous pairs of options and their choices, the trial order was randomized for delay × initial value with a unique order per participant. Because of the staircase approach, the subsequent trial was randomly chosen among 12 available pairs (6 delays × 2 initial values) including the pair that was present in the current trial. Again, the amount of delayed reward was determined by the aforementioned rule. The participants completed three blocks of the ITC task, 180 trials in total [(6 delays × 2 initial values × 5 steps) × 3 blocks].

#### Intervention tasks

Previous studies have suggested that episodic future thinking induces vivid imagination (simulation) of future reward delivery and, in turn, reduces reward delay discounting (22–24, 26). Inspired by this induction effect, we examined the impacts of two factors that are known to be linked with episodic future thinking (or mental stimulation) on impulsivity reduction: acquisition of additional context information and enhancement of individual working memory performance. Two intervention tasks were designed and used to examine these factors, along with three intervention tasks as controls. In the next section, we will explain the detailed designs of each intervention task.

#### Intervention task: Associative memory task

During the associative memory task, the participants were asked to memorize the associations between temporal delays and names of countries, which were introduced as pairs of information about the most wanted places to visit and the average duration people spend planning for the trip (Figure 1B and Supplementary Figure 1bi). This intervention was designed to implicitly provide additional information about delayed reward options. If additional context information benefits simulating delayed rewards, the intervention should decrease individuals’ delay discounting in the subsequent ITC task.

The associative memory task comprised two phases: the encoding and retrieval phases. Names of 30 different countries and 30 unique temporal delays (preparation time for the trip) were used as pairs (Supplementary Table 1). Among the pairs, only six levels of delays were shared with the delays in the ITC task (1, 10, 21, 50, 90, and 180 days; “target delays”), and the other 24 levels of delays (“non-target delays”) were never used in the ITC task. The names of the countries were sorted by their geographical distances from South Korea in ascending order, and they were matched with delays accordingly; examples include [Japan, 1 day], [Vietnam, 10 days], [Taiwan, 21 days], [The Philippines, 50 days], [New Zealand, 90 days], and [Brazil, 180 days]. During the encoding phase, there were 60 trials where 30 pairs that should be memorized were presented twice. In each trial, after the presentation of the association, the participants had to report whether they could successfully remember and vividly imagine the association between the paired pieces of information.

During the retrieval phase, the participants were asked to retrieve previously memorized association pairs and verbally report answers. Specifically, each retrieval trial was cued by one of the 30 delays, and participants’ verbal answers on the associated country names were recorded for the accuracy assessment. In each trial, the participants used a key press to notify that they finished providing the answer, and then they reported the extent to which they were confident about the answers on a 5-Likert scale (1 = not confident and 5 = very confident). The target delays were tested thrice, while the non-target delays were tested only once. In total, there were 42 trials (6 × 3 target cue trials + 24 non-target cue trials = 42) where the target and non-target delay cues were intermixed.

#### Intervention task: Control for the associative memory task (Control_AM_)

The procedures of the Control_AM_ task were largely identical to those of the associative memory task (Figure 1B and Supplementary Figure 1bii). The only difference was that for this control task, participants were asked to memorize the pairs of [country name, office supply item] instead of [country name, preparation time] (e.g., [Spain, Mouse controller]). Moreover, to match cognitive workload with the Associative memory task, office supplies items were used in a manner that word length of which names (in Korean) matched the number of digits of the delay it is replacing. We chose office supply items instead of delays so no cues during the ITC task may trigger retrieval of information regarding the learned association.

#### Intervention task: Working memory task

During the working memory task, the participants were asked to conduct a series of mental calculations (Figure 1C and Supplementary Figure 1ci). Specifically, the participants performed a task, the so-called “Serial 7s subtraction” (48) where they had to subtract seven from the answer in the previous trial. For example, if the answer in the previous trial was 200, the next answer that should be entered is 193. In the beginning, an initial number was randomly chosen between 107 and 999 and presented on the screen. The participants had to remember the most recently entered answer so they could use the information for the subsequent trial. Such need for reservation of information for a short duration is known to recruit participants’ working memory (49). There were three conditions where the task reset with a new initial number: when participants entered the wrong answer, when the answer for the subsequent trial was smaller than 100, or when participants did not enter an answer within 20 s. The participants completed two blocks of task where each block lasted for 10 min.

#### Intervention task: Control for the working memory task (Control_WM_)

The Control_WM_ task was designed to require continuous attention from participants but not working memory (Figure 1C and Supplementary Figure 1cii). In each trial, a circle-shaped stimulus showed up on either the left or right half of the screen, and the participants were instructed to press the left or right arrow key that matches the side of the screen. Stimulus color changed to yellow immediately after key press only if the key press was correct, and the color changed to gray when no answers were entered within 1.5 s, but no change happened when the submitted answer was incorrect. The next trial started after a short presentation of a feedback screen (0.5 s) and an inter-trial interval sampled from a uniform distribution [U(0.3; 0.7s)]. The participants completed two blocks of task where each block lasted for 10 min.

#### Intervention task: Resting

To examine the impacts of intervention tasks, all the participants went through two sessions of ITC tasks, one before and one after the corresponding intervention. To control for the possibility that impulsivity change may be induced by repeated participation in the ITC task, we introduced a resting phase for one of the five subgroups. The length of the resting phase was set to 20 min, matching the average length of other intervention tasks.

### Behavioral analyses

For each subgroup, participants’ tendency to choose the delayed option over the immediate option was calculated across three blocks of the ITC task separately for the task before and after an intervention task. As a model agnostic measure, the impact of each intervention was calculated as a difference in the choice ratio between the two ratio measures of choosing the immediate reward option over the delayed reward option. In addition, we estimated individuals’ delay discounting rate (*k*) based on participants’ choices across three blocks of the ITC task, separately for the task before and after an intervention. As noted above, we used the hyperbolic discount function SV = V/[1 + *k*D] (8–10, 45) and a softmax choice rule to explain the link between subjective valuation and choices as below:


$$\text{P(immediate option)}={\left(1+\exp\left[-\mu \left({\text{SV}}_{\text{immediate}}-{\text{SV}}_{\text{delayed}}\right)\right]\right)}^{-1}$$

where SV_immediate_ (SV_delayed_) is the subjective value of the immediate (delayed) reward option and μ is the inverse temperature, capturing the extent to which the individuals were sensitive to subjective value differences between the options. Note that the use of the hyperbolic value function has been shown useful in characterizing individuals’ impulsive (or patient) choice behaviors across a wide range of clinical populations including smokers, alcohol users, substance users, and pathological gamblers (2, 11, 13, 14, 19, 21, 41). We did not use alternative models and methods to quantify individual impulsivity (e.g., area under the empirically observed value function); it is out of the scope of this study to discuss the choice of valuation function and can be found elsewhere (20, 43, 47, 50). The delay discount rate *k* (0 < *k* < 1) and the inverse temperature μ (> 0) were set as individual-level free parameters such that best explaining parameter sets for each participant’s choice behaviors could be estimated. Individual parameter estimations were conducted with custom MATLAB R2017a scripts by maximum log-likelihood estimation (MLE) at the individual subject level. That is, based on the choice probability noted above, we maximized Σ_i=[1_,_2_,_…_,_180]_ log(P_i_), the sum of log probability of each participant’s choice in each trial. The *fminsearch* function in MATLAB with different initial values for each parameter estimation was used. After the parameter estimation, changes in delay discounting rates in each subgroup were used as model-based measures of the impact of the intervention tasks.

To examine whether the extent to which the individuals successfully completed their assigned intervention task was associated with change in behavioral choice patterns in the ITC task, the performance in the intervention tasks was measured accordingly. First, mean accuracies were calculated for each task. Second, for the working memory task and Control_WM_ task, temporal evolution in individuals’ performance was also examined. To do this, the tasks were binned into equal sizes (10 trials per bin) using a sliding window (5-trial overlap). We examined whether the participants showed increasing performance across bins, which would be an indication of successful training of the corresponding cognitive function. Furthermore, to better illustrate the impacts of intervention tasks on the changes in individuals’ impulsivity (i.e., delay discount rate), we divided each intervention task into five larger bins (each bin size was 1/3 of the entire task length where a sliding window was used with overlapping 1/6 of task length), and the association in each bin was calculated.

### Statistical analyses

The size of each intervention task group was relatively small; thus, any descriptive measures may be sensitive to a particular outlier. To prevent such biases, we used a bootstrapping method with 10,000 resampling iterations for within- and between- group mean comparisons. The *P*-value for each statistical analysis was calculated as the proportion of extreme samples (based on *t*-values) out of the entire resample estimates relative to the statistics estimated from the original data set. The same bootstrapping approach was used for regression analyses between intervention task performances and ITC behavioral patterns. In all the statistical tests, we used the delay discounting rate in log transformed form (log *k*), which is a commonly used method in statistical analyses of discounting rates due to their skewed distribution.

### Data and study material availability

The analytic scripts and data are available on GitHub.^1^

## Experiment 1

### Methods

In experiment 1, a total of 126 healthy individuals (male/female = 71/55, age = 22 ± 2.48) were recruited and randomly assigned to one of the five intervention tasks: two hypothesis-driven tasks, i) association memory task and ii) working memory task, and three control tasks (refer to section “Experimental procedures” for details). The associative memory task was designed to implicitly provide additional context information about delayed rewards (Figure 1B and Supplementary Figure 1b), and the working memory task was designed to boost individuals’ working memory capacity (Figure 1C and Supplementary Figure 1c). Specifically, by associative memory task intervention, we examined the possibility that the memorized association would be retrieved during the ITC task because of the use of common cues (i.e., delays for reward delivery vs. delays for planned trips; refer to section “Experimental procedures” for details), and that this additional information (i.e., planned trip locations) would provide contexts facilitating the simulation of future rewards (24). Using the working memory task, we tested the hypothesis of whether or not increase in working memory capacity would reduce one’s delay discounting. This hypothesis was constructed based on previous reports suggesting that mental calculation shares the same cognitive resources with time perception (51), which provides a crucial piece of information in future planning. Moreover, it is known that working memory serves to integrate information across multiple dimensions in mental simulation (38, 39). Thus, we expect that enhancing one’s working memory capacity may facilitate one’s future reward simulation.

Three control tasks comprised (i) another association task (Control_AM_) where the participants learned association about information irrelevant to delays (Figure 1B and Supplementary Figure 1b), (ii) a spatial attention task (Control_WM_) where participants were required of using continuous attention but not working memory (Figure 1C and Supplementary Figure 1c), and (iii) resting (refer to section “Experimental procedures” for task details). A second ITC task was administered to all the participants after each type of intervention, and the discount rate changes were calculated to examine the impacts of modulating each factor on reducing individuals’ delay discounting. The participants were paid at the end of the study based on the delay and reward of a single random choice drawn from all the ITC task decisions the participants made.

Twenty-six students participated in the associative memory task, and 24 students participated in the working memory task. For the control tasks, a comparable number of students were assigned who were age-, sex-, and education-matched [control for associative memory task (Control_AM_): *N* = 25; control for working memory task (Control_WM_): *N* = 24; resting: *N* = 26]. From the entire participant pool, we excluded six participants from the analyses because they explicitly expressed that they would rather like to receive class credits than monetary rewards as compensation for participation; we considered this as an indication of different, subjective incentive structure and thus excluded these participants. One additional participant was excluded from the analyses because he showed up at the laboratory under the influence (drunk). Additional eight participants were excluded for whom the individual-level computational model (refer to section “Behavioral analyses”) did not come up with a unique solution by maximum likelihood estimation (MLE). After the exclusion, data from 111 participants (male/female = 60/51, age = 22.07 ± 2.54) were used for the analyses (see Table 1 for demographic information for each subgroup).

**TABLE 1:** Demographic data for experiment 1.

	Associative memory (*N* = 23)	Control_AM_ (*N* = 22)	Working memory (*N* = 22)	Control_WM_ (*N* = 21)	Resting (*N* = 23)
Age (years)^a^	22.22 ± 2.71	21.45 ± 2.15	22.32 ± 2.44	22.57 ± 2.96	21.83 ± 2.46
Male/female^b^	11/12	12/10	17/5	11/10	9/14
**Education level^c^**					
Some college	17	19	19	15	18
Finished college	6	3	3	6	5

Five subgroups were of comparable age^a^ [*F*(4, 106) = 0.64, *P* = 0.63], gender^b^ [χ^2^(4) = 5.08, *P* = 0.28], and education^c^ [χ^2^(4) = 2.55, *P* = 0.64].

### Results

**FIGURE 2:**
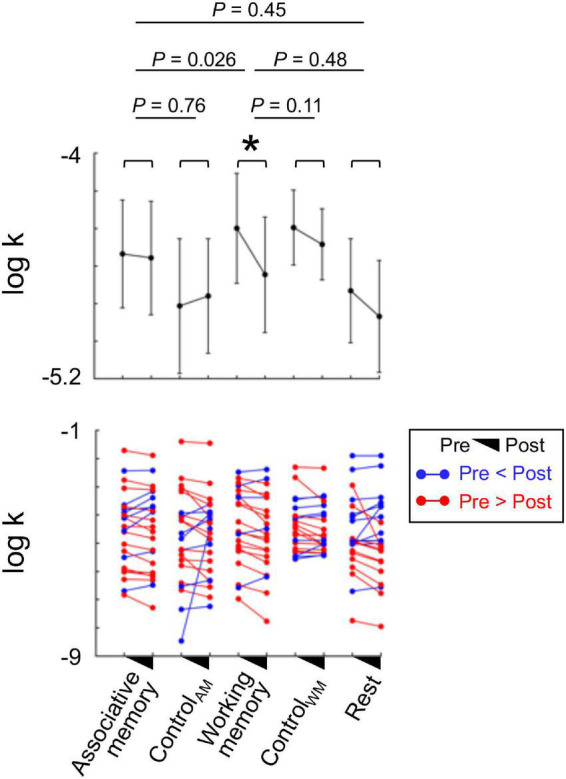
Impacts of the intervention tasks on reducing individuals’ impulsivity (experiment 1). Each participant group was given a different type of intervention task. To examine whether each intervention had an impact on individuals’ impulsivity, we calculated changes in individuals’ delay discount rates (*k*) by comparing their behavioral patterns during the intertemporal choice (ITC) task conducted before and after the intervention task. The individuals showed significantly reduced impulsivity after participating in the working memory task [paired *t*-test, *t*(20) = 3.44, *P* = 0.0026, Cohen’s d = 0.75, bootstrapping *P* = 0.0031]. On the contrary, the individuals who participated in other intervention tasks did not show the same pattern (i.e., reduced impulsivity) (all bootstrapping *p*s > 0.05). The mean discount rate change in the individuals who participated in the working memory task was larger compared with that in the individuals who were assigned to the associative memory task in between two ITC tasks [independent-sample *t*-test, *t*(42) = –2.31, *P* = 0.026, Cohen’s d = –0.35, bootstrapping *P* = 0.026]. The impact of the working memory task was not statistically different from that of the corresponding control task (Control_WM_; bootstrapping *P* = 0.11) or the resting (bootstrapping *P* = 0.48). Each dot represents an individual participant, and error bars indicate s.e.m.; *P < 0.05.

All the participants completed the first assessment of the ITC task to have their individual baseline discount rates estimated (log-transformed *k*; refer to section “Behavioral analyses” for parameter estimation procedures). In the first assessment, the five subgroups showed comparable baseline delay discount rates [*F*(4, 105) = 0.43, *P* = 0.78, one-way ANOVA; Figure 2]. This allowed us to define the impact of each intervention as the behavioral differences between pre- and post- intervention tasks; we calculated Δlog *k* = log (*k*_pre–intervention_)–log (*k*_post–intervention_) for model-based analyses where *k* indicates an individual’s estimated discount rate (larger *k* refers to more impulsive behavioral choices). In the measure Δlog *k*, positive numbers indicate a positive intervention effect on enhancing preference for the delayed reward option (i.e., reducing impulsivity).

As explained above, choices from individuals who were assigned to the associative memory task and to the working memory task were used to test the impacts of the context information and the working memory capacity on reducing impulsivity, respectively (Figure 2). The mixed-design ANOVA setting “Time” (pre- and post- intervention) as a within-group factor and “Type”(associative memory, working memory, and rest) as a between-group factor revealed a significant main effect of time [*F*(1, 64) = 5.79, *P* = 0.019]. However, the main effect of type and the interaction of time × type were not significant [type: *F*(2, 64) = 0.28, *P* = 0.75; interaction: *F*(2, 64) = 1.39, *P* = 0.26], suggesting that neither intervention task had superior efficiency in reducing individuals’ impulsivity compared to simple repetition of the ITC (i.e., the rest group). We suspected a possibility that the impact of each intervention may be tightly intertwined with their performances in the cognitive intervention task and further examined this possibility to explore the impacts of potential intervention mechanisms.

Within each intervention group, the working memory task was the only intervention that had a significant impact on two ITC measures such that the individuals showed a significantly reduced temporal discounting rate after the intervention [paired *t*-test, *t*(20) = 3.44, *P* = 0.0026, Cohen’s *d* = 0.75, bootstrapping *P* = 0.0031; Figure 2]. The individuals who took part in the associative memory task as an intervention and who rested between two measures of ITC all did not show a difference in their estimated temporal discounting rate [associative memory: *t*(22) = 0.34, *P* = 0.74, Cohen’s d = 0.07, bootstrapping *P* = 0.74; Rest: t(22) = 1.049, *P* = 0.31, Cohen’s d = 0.22, bootstrapping *P* = 0.31; Figure 2]. Note that neither of the two additional tasks (Control_WM_ and Control_AM_), each of which was examined as a control for the working memory and associative memory interventions, respectively, was effective in reducing individuals’ delay discounting rates [Control_WM_: t(20) = 1.4, *P* = 0.18, Cohen’s d = 0.31, bootstrapping *P* = 0.19; Control_AM_: t(21) = –0.25, *P* = 0.8, Cohen’s d = –0.054, bootstrapping *P* = 0.81].

Direct comparisons between the intervention groups showed that the reduction in delay discounting rate in the working memory group was significantly larger than that in the associative memory group [two-sample *t*-test, *t*(42) = 2.31, *P* = 0.026, Cohen’s d = 0.35, bootstrapping *P* = 0.026; Figure 2]. Although trending toward the superior impact, the impact of the working memory intervention was comparable with that of Control_WM_ [*t*(40) = 1.65, *P* = 0.11, Cohen’s d = 0.25, bootstrapping *P* = 0.11]and that of rest [*t*(42) = 0.73, *P* = 0.47, Cohen’s d = 0.11, bootstrapping *P* = 0.48; Figure 2]. The impact of the associative memory intervention was also comparable with that of its corresponding control tasks [associative memory vs. Control_AM_: *t*(43) = 0.35, *P* = 0.73, Cohen’s d = 0.052, bootstrapping *P* = 0.76; vs. Rest: *t*(44) = –0.78, *P* = 0.44, Cohen’s d = –0.12, bootstrapping *P* = 0.45; Figure 2]. These results all together suggest that individuals’ working memory capacity, but not abundance of context information or individuals’ attention level, is linked with their preference for delayed rewards. Yet again, given the statistically comparable impacts of the intervention tasks, one should be careful not to overinterpret the involvement of working memory in the simulation of future rewards.

For completeness, we also compared the impacts of each intervention design using model-agnostic measures; ΔP(immediate option) = P(immediate option_pre–intervention_)–P(immediate option_post–intervention_) was calculated as a model-agnostic measure where P(immediate option) refers to the probability of choosing the immediate over the delayed reward option. Note that the model-based measure, delay discounting rate *k*, is more appropriate in quantifying individuals’ characteristics of future reward simulation, because the model-agnostic measure cannot tease apart the character of interest (i.e., delay discounting rate) from a confounding factor (e.g., value sensitivity, denoted as μ; refer to 2.3. Behavioral analyses). Nevertheless, the statistical results were largely the same as model-based results (refer to Supplementary Figure 2 for statistical results).

**FIGURE 3:**
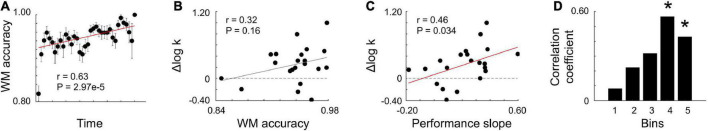
Individual performance enhancement in working memory training is associated with level of impulsivity reduction (experiment 1). **(A)** Change over time in working memory task accuracy was measured to examine whether or not the individuals showed performance enhancement. Average accuracy within each 10-trial sliding window (with 5-trial overlap) was calculated per participant. Indeed, the participants showed a significant performance improvement over time (Pearson’s correlation *r* = 0.64, *P* = 2.97e-5). Each dot represents mean accuracy within each window across participants, and error bars indicate s.e.m. **(B)** Although statistically not significant, individuals’ overall performance (i.e., accuracy) in the working memory task had a trend of positive correlation with the changes in their delay discounting rates (*r* = 0.32, *P* = 0.16, bootstrapping *P* = 0.16). Specifically, individuals who had the highest (lowest) accuracy in the working memory task showed a relatively larger (smaller) reduction in their estimated impulsivity (positive Δlog *k* indicates one’s impulsivity reduction). **(C)** Independent of individuals’ initial accuracy level, individuals’ speed of working memory performance enhancement (performance slope) was significantly correlated with level of impulsivity reduction (*r* = 0.46, *P* = 0.036, bootstrapping *P* = 0.034). Each dot represents an individual, and solid red lines are robust regression lines. **(D)** By dividing the working memory task into five bins (bin size = 1/3 of the entire task length, sliding window with 1/6 overlap), a stark difference was observed between bins in the association between individuals’ working memory accuracy and their impulsivity reduction levels. Particularly, individuals’ working memory task performance in the later period (fourth and fifth bins) showed strong associations with the extent to which they make less impulsive choices in the post-intervention ITC task. *P < 0.05.

We further examined whether or not individuals’ performance in the working memory task is associated with the level of impulsivity reduction. Repetition of working memory tasks typically results in improvement of individuals’ task performance, which is considered a signature of cognitive training (41, 43). Although our working memory task as a type of intervention task was only in the order of tens of minutes rather than days as in other cognitive training studies, we also observed such a performance enhancement across time (Figure 3A). Specifically, 91% of the participants showed performance increase along the task and across individuals, the proportion of correct answers significantly increased along the course of the task (*r* = 0.64, *P* = 2.97e-5). To examine the impact of this intervention task on individuals’ impulsivity, we examined whether or not the average accuracy and/or the speed of performance improvement was associated with the change in delay discount rate.

There was a positive but statistically non-significant trend between average working memory performance and reduction in delay discounting (Pearson correlation, *r* = 0.32, *P* = 0.16, bootstrapping *P* = 0.16; Figure 3B). On the contrary, participants who had steeper improvement in the working memory performance showed a significantly larger reduction in their impulsivity (*r* = 0.46, *P* = 0.036, bootstrapping *P* = 0.034; Figure 3C). This result was corroborated by the association between impulsivity reduction and average working memory performance in each quintile of the entire task such that only the last two phases showed their significant association (bin 1: *r* = 0.081, *P* = 0.73; bin2: *r* = 0.22, *P* = 0.33; bin 3: *r* = 0.32, *P* = 0.16; bin 4, *r* = 0.57, *P* = 0.0074; bin 5, *r* = 0.43, *P* = 0.051; Figure 3D). These results suggest that independent of individuals’ initial working memory capacity, how fast and to what extent individuals get trained for their working memory capacity may be associated with their behavioral preference for delayed rewards. Refer to Supplementary Figure 3 for the correlation results between performances in the other intervention tasks and individuals’ impulsivity changes.

There was a stark difference in individuals’ behavioral patterns during the Control_WM_ task (i.e., spatial attention task). Across the task, individuals’ task performance (accuracy) gradually decreased (Pearson correlation, *r* = –0.36, *P* = 0.0011, Supplementary Figure 4D); 66.67% of the participants showed decreasing task performance over time. Given this pattern, it is tempting to interpret the results as evidence that participants experienced more mental fatigue over an easy and laborious task (52) than being trained for a specific type of executive function (e.g., selective attention). However, we cannot neglect the alternative possible explanation that participants’ task performance reflects a ceiling effect due to low task difficulty, which introduces a potential bias toward finding a decrement effect. Nevertheless, we directly examined the impact of the task on individuals’ impulsivity and found that neither the mean task accuracy nor the performance slope (estimated speed of task performance change) of Control_WM_ was correlated with the extent to which individuals’ delay discounting decreased (Supplementary Figures 3d, 4e,f). Together, there was no clear evidence suggesting that participating in a continuous attention task has an effect on reducing individuals’ impulsivity.

## Experiment 2

### Methods

Experiment 2 was a follow-up study that was designed to test whether or not the results from the first experiment could be replicated. The participants were randomly assigned to one of the three intervention tasks: the working memory task and two control tasks. Thirty students participated in the working memory task. For the control tasks, a comparable number of students, whose age, sex, and education were matched, were assigned (Control_WM_: *N* = 30; Resting: *N* = 30). Eleven participants were excluded from the analysis because the individual-level computational model did come up with a unique solution by MLE (refer to 2.3. Behavioral analyses). In addition, nine participants who had discount rate estimates larger or smaller than 3 median absolute deviations (MAD) were excluded as outliers. After the exclusion, data from 70 participants (male/female = 41/29, age = 22.17 ± 4.08) were used for the analyses (refer to Table 2 for demographic information on each subgroup).

**TABLE 2:** Demographic data for experiment 2.

	Working memory (*N* = 25)	Control_WM_ (*N* = 26)	Resting (*N* = 19)
Age (years)^a^	22.64 ± 5.53	20.77 ± 2.47	22.16 ± 2.95
Male/female^b^	13/12	15/11	13/6
**Education level^c^**			
Some college	16	23	14
Finished college	9	3	5

Five subgroups were of comparable age^a^ [*F*(2, 67) = 3.37, *P* = 0.04], gender^b^ [χ^2^(2) = 1.21, *P* = 0.55], and education^c^ [χ^2^(2) = 4.21, *P* = 0.12].

### Results

**FIGURE 4:**
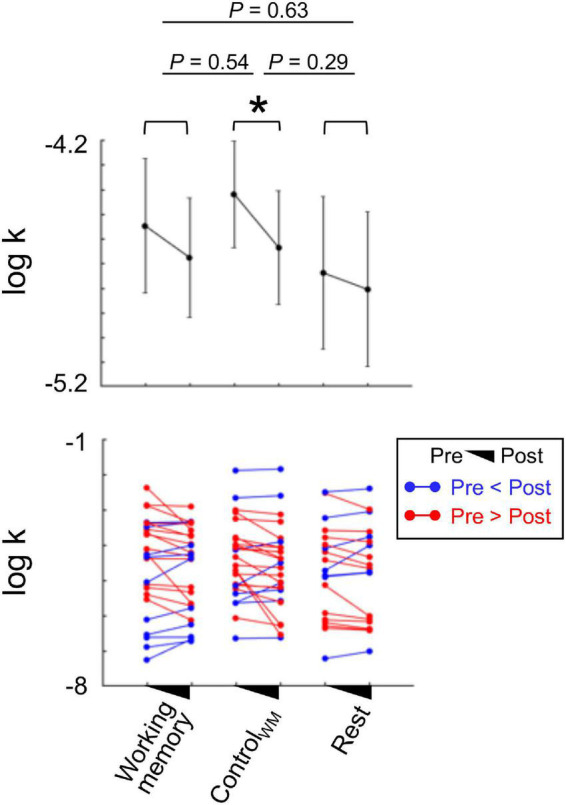
Impacts of the intervention tasks on reducing individuals’ impulsivity (experiment 2). In experiment 2, three types of intervention task (working memory, Control_WM_, and rest) were tested. To examine whether each intervention had an impact on individuals’ impulsivity, we calculated changes in individuals’ delay discount rates (*k*) by comparing their behavioral patterns during the ITC task conducted before and after the intervention task. The individuals showed significantly reduced impulsivity after participating in the Control_WM_ task [paired *t*-test, *t*(20) = 2.07, *P* = 0.049, Cohen’s d = 0.41, bootstrapping *P* = 0.051]. On the contrary, the individuals who participated in the other intervention tasks including the working memory task did not show the same pattern (all bootstrapping *p*s > 0.05). The mean discount rate change in individuals who participated in the Control_WM_ task was not larger compared with that in individuals with any intervention task (all bootstrapping *p*s > 0.05). Each dot represents an individual participant, and error bars indicate s.e.m.; *P < 0.05.

In experiment 1, we observed partial evidence suggesting that working memory enhancement may reduce individuals’ impulsivity. To further examine whether the results regarding the effect of working memory training on impulsivity reduction are replicable, we conducted an additional independent experiment where 70 participants (male/female = 41/29, age = 22.17 ± 4.08; no overlap with experiment 1) were asked to perform either the working memory or the spatial attention task (Control_WM_), or to rest between two assessments of their impulsivity. As in experiment 1, the three sub-groups showed comparable delay discount rates [*F*(2, 67) = 0.35, *P* = 0.71, one-way ANOVA; Figure 4], which indicates that individuals’ baseline impulsivity levels were matched across groups.

We analyzed participants’ choice data following the same protocol used in experiment 1. Individuals’ discount rates (*k*) were estimated from their behavioral choices as their impulsivity measure, and we used Δlog *k* as an indication of the impacts of intervention task. First, we tested the impacts of intervention tasks on individuals’ impulsivity reduction. Consistent with experiment 1, we observed a significant main effect of time [pre- vs. post- intervention; *F*(1, 67) = 6.81, *P* = 0.011], but no significant effect of type [working memory, Control_WM_, and Rest; *F*(2, 67) = 0.22, *P* = 0.81] or interaction of time × type [*F*(2, 67) = 0.59, *P* = 0.56]. The model agnostic analyses of individuals’ choices were largely the same as the model-based results (refer to Supplementary Figure 5 for statistical results). The results of both experiments indicate that there is a significant repetition effect on reducing individuals’ impulsivity, and that there is no evidence to support the impact of intervention tasks.

Examining individuals’ impulsivity changes (changes in discount rates) between two assessments within each intervention group showed inconsistent patterns compared with experiment 1. Specifically, the group who took part in the working memory task or rested did not show a significant effect of the intervention task [working memory: paired *t*-test, *t*(25) = 1.37, *P* = 0.18, Cohen’s d = 0.27, bootstrapping *P* = 0.18; rest: *t*(19) = 0.86, *P* = 0.39, Cohen’s d = 0.2, bootstrapping *P* = 0.4], while the Control_WM_ group showed a significant reduction in their impulsivity [*t*(26) = 2.07, *P* = 0.047, Cohen’s d = 0.41, bootstrapping *P* = 0.048]. Given these inconsistencies, one may suspect that our null results regarding the type of intervention task are due to a lack of power. However, this is unlikely given that the results hold consistent even when we collapsed the data from both experiments [time: *F*(1, 125) = 13.11, *P* = 4.25e-04; type: *F*(2, 125) = 0.27, *P* = 0.77; time × type: *F*(2, 125) = 0.15, *P* = 0.86].

**FIGURE 5:**
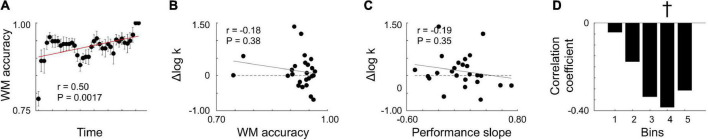
Individual performance enhancement in working memory training is not associated with level of impulsivity reduction (experiment 2). **(A)** The participants showed a significant performance improvement in the working memory task over time (Pearson’s correlation *r* = 0.50, *P* = 0.0017). Each dot represents mean accuracy within each window across participants, and error bars indicate s.e.m. **(B)** Individuals’ overall working memory performance was not associated with changes in their delay discounting rates (*r* = –0.18, *P* = 0.38, bootstrapping *P* = 0.36). **(C)** Individuals’ speed of working memory performance enhancement (performance slope) was not correlated with level of impulsivity reduction (*r* = –0.19, *P* = 0.35, bootstrapping *P* = 0.35). Each dot represents an individual, and solid red lines are robust regression lines. **(D)** Over the course of the working memory task, no significant association was observed between individuals’ working memory task accuracy and their impulsivity reduction level; if any, there were negative trends that showed that the individuals tended to show more impulsive choices in the post-intervention ITC task. ^†^< 0.1.

Second, we examined the association between individuals’ working memory task performances and their levels of impulsivity reduction. Note that consistent with experiment 1, the individuals showed performance increases along the course of the working memory task (*r* = 0.5, *P* = 0.0017; Figure 5A). In contrast to the association observed in the first experiment, neither the average accuracy in the working memory task (*r* = –0.18, *P* = 0.38; Figure 5B) nor the speed of performance improvement (*r* = –0.19, *P* = 0.35; Figure 5C) showed a significant correlation with the change in individuals’ discount rate. Moreover, the gradual increase in the association between task improvement and impulsivity reduction, which we interpreted as evidence of the impact of the intervention task, disappeared in the replication data (Figure 5D). The association between individuals’ performances in the Control_WM_ task and their impulsivity changes was also examined, albeit the results did not provide any clear evidence suggesting its impact on impulsivity reduction as reported in experiment 1 (Supplementary Figures 6b, 7e,f).

## Discussion

Impulsive behavioral tendency is a typical hallmark of individuals who show health risk behaviors such as substance use and cigarette smoking, and has been a plausible target for interventions (12). The current study focused on two potential cognitive factors that are closely linked to successful future simulation and examined their impacts on modulating individuals’ impulsivity. By conducting two independent experiments, we found a significant reduction in individuals’ impulsivity in the second assessment compared to their original impulsivity regardless of the type of intervention task administered between two ITC tasks. However, there was no consistent evidence supporting that either of the cognitive intervention tasks has a specific and reliable impact on reducing individuals’ impulsivity.

Mental simulation is suggested as one of the four modes of future thinking (53), and it is broadly defined as the cognitive construction of hypothetical scenarios or the reconstruction of real events. As a high-level cognitive function, future thinking is known to be crucial in planning, decision-making, and learning (46, 54–59). Recent studies showed that future thinking also plays an important role in evaluating temporally delayed rewards (22, 24). Specifically, individuals preferred delayed rewards more than they used to after being enforced to focus on episodic future thoughts (24). Given the well-known close relationship between individuals’ health risk behaviors in life and their impulsive choice behaviors in laboratory tasks, we examined two potential factors that may underlie or be related to episodic future thinking to explore and evaluate the impacts of simple cognitive training tasks as potential interventions in reducing individuals’ impulsivity. By training and enhancing cognitive abilities related to each mechanism, we expected individuals’ mental simulation to become more fluent and, in turn, contribute to impulsivity reduction. In contrast to our expectation, across two independent tasks, we did not find any specific modulation effects from either of the targeted cognitive intervention tasks. These results may be due to the complex nature of the future-oriented thinking process that cannot be separated into independent components. Thus, each type of intervention tested in the current study, without being paired up with other complementary tasks, did not have either strong or reliable modulatory effects.

In previous studies, the impacts of working memory training on reducing individuals’ impulsivity have been inconclusive. Although the intervention task we tested in the current study (i.e., Serial 7s subtraction) is not a conventional working memory training task, it was selected to maximize the active engagement of participants in our gamified intervention task (60). Indeed, as we expected, most of the participants successfully showed an increased performance across the brief training session. However, our two independent experiments showed mixed results regarding impulsivity reduction. Given the short training period that our study implemented, we do not claim that all types of working memory training are ineffective in impulsivity reduction. Future studies may systematically examine the dose effects of working memory enhancement on individuals’ impulsivity reduction.

Taking two independent experimental data together, individuals’ the reduced impulsivity observed in the current study was not linked to a specific type of intervention task but rather appeared as a general phenomenon. The results suggest that our study design may be triggering cognitive and affective mechanisms over the repeated applications of ITC tasks. Such malleability of intertemporal choice has been documented across various contexts (61). For example, attending to a particular type of information (e.g., magnitude of rewards) or updating one’s expectations based on recent experiences are known to affect individuals’ impulsivity (62–64). In the current study, the participants already went through entire series of choices between delayed and immediate options before the intervention tasks, which could have affected individuals’ expectations about the reward delays. The participants perceived reward delays as less distant when expecting non-zero days of delay as a new “reference” delay (64–66) in the beginning of the second assessment and, in turn, made less impulsive choices. We unfortunately did not collect a direct measure of individuals’ expected delays; thus, our suggested interpretation needs further investigation.

There are a few alternative explanations for why the individuals showed reduced impulsivity in the second assessment independent of the type of intervention task. First, because the individuals already tried simulating future rewards once, simulation of the value of delayed rewards might have been facilitated. Previously, it has been shown that individuals who could vividly project their previous experience into the future showed a particularly large impulsivity reduction from episodic future thinking (24). Our intervention tasks between two ITC tasks lasted around 20 min, and we cannot fully rule out the possibility that the individuals remembered their first choices and viewed options, albeit the choice sequences were randomized. Note that this possibility should be considered apart from a simple practice effect, because there was no evidence of impulsivity reduction along three repeated blocks within each experiment [experiment 1: *F*(2, 581) = 1.78, *P* = 0.17; experiment 2: *F*(2, 415) = 0.52, *P* = 0.6]. Second, the participants might have felt bored or stressed out in the later stage of the experiment. Besides cognitive context manipulations, being under specific emotional states is also known to make individuals more or less impulsive (67, 68). Third, letting the individuals spend a period of time participating in the other types of cognitive tasks (or mind wondering) might have led them to think about thoughts unrelated to delayed rewards and had an impact on reducing delay discounting (69, 70). Future studies can be tailored to directly test each hypothesis and disambiguate the mechanisms explaining why individuals’ impulsivity decreases in a repeat assessment.

The current study has some limitations that need further investigation. First, the null results from the second experiment might be due to the small sample size and lack of statistical power. This is unlikely to be the main factor, because the results regarding the impact of working memory training did not hold when both data sets from the two experiments were pooled together. Second, there is a possibility that the individuals are able to access and use a mental number line in memorizing the place-time association. If that is the case, it means that the associated place information is irrelevant to the “future thinking” process required during the delay discounting task, and this could be why the association task did not have an impact on individuals’ impulsivity. Third, we only examined the impacts of fixed doses of intervention tasks; thus, we cannot completely rule out that a larger dose (longer training and repetition) would induce significant impulsivity reduction effects. Fourth, in contrast to the first experiment, the second experiment was conducted during the COVID-19 pandemic (experiment 1: 25 March 2019–20 May 2019; experiment 2: 13 January 2021–19 July 2021), and individuals, in general, are expected to be in negative mental states (e.g., high stress, depression, and anxiety) (71, 72). Given the close relationship between the ongoing pandemic and health risk behaviors (73–75), we cannot overlook the possibility that the null effect of intervention training in the second experiment is associated with environmental changes and accompanying changes in mental states.

Nevertheless, the current study taps into two possible cognitive mechanisms that are linked to future reward simulation and evaluates their potential effectiveness in intervening in individuals’ delay discounting behavior. Based on the two independent experiments, no specific task among the examined intervention tasks showed a reliable impact on reducing individuals’ impulsivity. Although our results do not directly inform why other previous studies using episodic future thinking as an intervention method reported mixed results (31, 32), our negative findings again call attention to the importance of mechanistic understanding of suggested cognitive training and intervention designs (76). Rather unexpectedly, we observed a type-general impulsivity reduction effect, which could not be explained as a simple task learning effect. It is tempting to conclude that a simple repetition of ITC may have “nudged” people to think (and simulate) more about future anticipated rewards. If so, recurring examination of individuals’ impulsivity could be the only and simplest intervention design one needs to achieve impulsivity reduction. However, future studies should explore more on this possibility; how many times should the task be repeated (i.e., dose-effect)? How long would an individual’s reduced impulsivity last? Would this change in impulsivity being transferred to individuals’ real-life health risk behavior?

The current study leaves an open question on how to design cognitive training paradigms that target more specific cognitive functions for effective and efficient intervention for individuals who have health risk problems such as obesity (77–79), substance addiction (12, 80, 81), and pathological gambling (13, 82). Still, the results point out once more that an individual’s tendency to act impulsively may not be a trait-like attribute that does not change but a malleable feature that can be changed differently depending on situated contexts [e.g., education, and socioeconomic status of the family (83)].

## Data availability statement

The datasets presented in this study can be found in online repositories. The names of the repository/repositories and accession number(s) can be found below: https://github.com/dongilchung/impulsivity-intervention.

## Ethics statement

The studies involving human participants were reviewed and approved by the Institutional Review Boards of Ulsan National Institute of Science and Technology. The patients/participants provided their written informed consent to participate in this study.

## Author contributions

MH, S-PK, and DC designed the experiments. MH and DC analyzed the data and drafted the manuscript. All authors discussed the results and revised and approved the final version of the manuscript.

## Footnotes

1
https://github.com/dongilchung/impulsivity-intervention

## References

[1] Ainslie G. Specious reward: a behavioral theory of impulsiveness and impulse control. *Psychol Bull.* (1975) 82:463. 10.1037/h0076860 1099599

[2] Baker F Johnson MW Bickel WK. Delay discounting in current and never-before cigarette smokers: similarities and differences across commodity, sign, and magnitude. *J Abnorm Psychol.* (2003) 112:382. 10.1037/0021-843X.112.3.382 12943017

[3] Bickel WK Marsch LA. Toward a behavioral economic understanding of drug dependence: delay discounting processes. *Addiction.* (2001) 96:73–86. 10.1046/j.1360-0443.2001.961736.x 11177521

[4] Kable JW Glimcher PW. The neural correlates of subjective value during intertemporal choice. *Nat Neurosci.* (2007) 10:1625–33. 10.1038/nn2007 17982449 PMC2845395

[5] Figner B Knoch D Johnson EJ Krosch AR Lisanby SH Fehr E Lateral prefrontal cortex and self-control in intertemporal choice. *Nat Neurosci.* (2010) 13:538–9. 10.1038/nn.2516 20348919

[6] Hare TA Camerer CF Rangel A. Self-control in decision-making involves modulation of the vmPFC valuation system. *Science.* (2009) 324:646–8. 10.1126/science.1168450 19407204

[7] McClure SM Laibson DI Loewenstein G Cohen JD. Separate neural systems value immediate and delayed monetary rewards. *Science.* (2004) 306:503–7. 10.1126/science.1100907 15486304

[8] Frederick S Loewenstein G O’donoghue T. Time discounting and time preference: A critical review. *J Econ Lit.* (2002) 40:351–401. 10.1257/jel.40.2.351

[9] Green L Myerson J. A discounting framework for choice with delayed and probabilistic rewards. *Psychol Bull.* (2004) 130:769–92. 10.1037/0033-2909.130.5.769 15367080 PMC1382186

[10] Mazur JE. An adjusting procedure for studying delayed reinforcement. In: Commons ML Mazur JE Nevin JA Rachlin H editors. *The Effect of Delay And of Intervening Events on Reinforcement Value.* Mahwah, NJ: Lawrence Erlbaum Associates, Inc (1987). p. 55–73.

[11] Grosskopf CM Kroemer NB Pooseh S Böhme F Smolka MN. Temporal discounting and smoking cessation: choice consistency predicts nicotine abstinence in treatment-seeking smokers. *Psychopharmacology.* (2021) 238:399–410. 10.1007/s00213-020-05688-5 33216166 PMC7826310

[12] MacKillop J Amlung MT Few LR Ray LA Sweet LH Munafo MR. Delayed reward discounting and addictive behavior: a meta-analysis. *Psychopharmacology (Berl).* (2011) 216:305–21. 10.1007/s00213-011-2229-0 21373791 PMC3201846

[13] Dixon MR Marley J Jacobs EA. Delay discounting by pathological gamblers. *J Appl Behav Anal.* (2003) 36:449–58. 10.1901/jaba.2003.36-449 14768665 PMC1284461

[14] Petry NM Casarella T. Excessive discounting of delayed rewards in substance abusers with gambling problems. *Drug Alcohol Depend.* (1999) 56:25–32. 10.1016/S0376-8716(99)00010-1 10462089

[15] Cheng Y-S Ko H-C Sun C-K Yeh P-Y. The relationship between delay discounting and Internet addiction: A systematic review and meta-analysis. *Addict Behav.* (2021) 114:106751. 10.1016/j.addbeh.2020.106751 33310692

[16] Bickel WK Wilson AG Franck CT Mueller ET Jarmolowicz DP Koffarnus MN Using crowdsourcing to compare temporal, social temporal, and probability discounting among obese and non-obese individuals. *Appetite.* (2014) 75:82–9. 10.1016/j.appet.2013.12.018 24380883 PMC3998832

[17] Story G Vlaev I Seymour B Darzi A Dolan R. Does temporal discounting explain unhealthy behavior? A systematic review and reinforcement learning perspective. *Front Behav Neurosci.* (2014) 8:76. 10.3389/fnbeh.2014.00076 24659960 PMC3950931

[18] Amlung M Marsden E Holshausen K Morris V Patel H Vedelago L Delay discounting as a transdiagnostic process in psychiatric disorders: a meta-analysis. *JAMA Psychiatry.* (2019) 76:1176–86. 10.1001/jamapsychiatry.2019.2102 31461131 PMC6714026

[19] Coffey SF Schumacher JA Baschnagel JS Hawk LW Holloman G. Impulsivity and risk-taking in borderline personality disorder with and without substance use disorders. *Pers Disord Theory Res Treat.* (2011) 2:128. 10.1037/a0020574 22448732

[20] Garcia-Perez A Weidberg S González-Roz A Alonso-Perez F Secades-Villa R. Relationship between delay discounting and depression in cigarette smokers and non-smokers. *Addict Behav.* (2020) 103:106251. 10.1016/j.addbeh.2019.106251 31874376

[21] Maraz A Andó B Rigó P Harmatta J Takách G Zalka Z The two-faceted nature of impulsivity in patients with borderline personality disorder and substance use disorder. *Drug Alcohol Depend.* (2016) 163:48–54. 10.1016/j.drugalcdep.2016.03.015 27107850

[22] Benoit RG Gilbert SJ Burgess PW. A neural mechanism mediating the impact of episodic prospection on farsighted decisions. *J Neurosci.* (2011) 31:6771–9. 10.1523/JNEUROSCI.6559-10.2011 21543607 PMC6632845

[23] Hakimi S Hare TA. Enhanced neural responses to imagined primary rewards predict reduced monetary temporal discounting. *J Neurosci.* (2015) 35:13103–9. 10.1523/JNEUROSCI.1863-15.2015 26400940 PMC6605429

[24] Peters J Büchel C. Episodic future thinking reduces reward delay discounting through an enhancement of prefrontal-mediotemporal interactions. *Neuron.* (2010) 66:138–48. 10.1016/j.neuron.2010.03.026 20399735

[25] Rösch SA Stramaccia DF Benoit RG. Promoting farsighted decisions *via* episodic future thinking: A meta-analysis. *J Exp Psychol Gen.* (2022) 151:1606. 10.1037/xge0001148 34843367

[26] Schacter DL Addis DR Hassabis D Martin VC Spreng RN Szpunar KK. The future of memory: remembering, imagining, and the brain. *Neuron.* (2012) 76:677–94. 10.1016/j.neuron.2012.11.001 23177955 PMC3815616

[27] Roberts A Rogers J Mason R Siriwardena AN Hogue T Whitley GA Alcohol and other substance use during the COVID-19 pandemic: A systematic review. *Drug Alcohol Depend.* (2021) 229:109150. 10.1016/j.drugalcdep.2021.109150 34749198 PMC8559994

[28] Kleykamp BA Guille C Barth KS McClure EA. Substance use disorders and COVID-19: the role of telehealth in treatment and research. *J Soc Work Pract Addict.* (2020) 20:248–53. 10.1080/1533256X.2020.1793064

[29] Prochaska JJ Vogel EA Chieng A Baiocchi M Maglalang DD Pajarito S A randomized controlled trial of a therapeutic relational agent for reducing substance misuse during the COVID-19 pandemic. *Drug Alcohol Depend.* (2021) 227:108986. 10.1016/j.drugalcdep.2021.108986 34507061 PMC8423936

[30] Prochaska JJ Vogel EA Chieng A Kendra M Baiocchi M Pajarito S A therapeutic relational agent for reducing problematic substance use (Woebot): development and usability study. *J Med Internet Res.* (2021) 23:e24850. 10.2196/24850 33755028 PMC8074987

[31] Aonso-Diego G González-Roz A Martínez-Loredo V Krotter A Secades-Villa R. Episodic future thinking for smoking cessation in individuals with substance use disorder: Treatment feasibility and acceptability. *J Subst Abuse Treat.* (2021) 123:108259. 10.1016/j.jsat.2020.108259 33612193

[32] Voss AT Jorgensen MK Murphy JG. Episodic future thinking as a brief alcohol intervention for heavy drinking college students: A pilot feasibility study. *Exp Clin Psychopharmacol.* (2022) 30:313. 10.1037/pha0000451 33630649

[33] Rö,sch SA Stramaccia DF Benoit RG. Promoting farsighted decisions *via* episodic future thinking: A meta-analysis. *J Exp Psychol Gen.* (2021) 151:1606. 10.31234/osf.io/53ju2 34843367

[34] D’Argembeau A Ortoleva C Jumentier S Van der Linden M. Component processes underlying future thinking. *Mem Cogn.* (2010) 38:809–19. 10.3758/MC.38.6.809 20852243

[35] Chang Y-C Ladd BO. Effects of content and valence of episodic future thinking on delay discounting and alcohol demand. *Psychol Addict Behav.* (2022) Advance Online Publication. 10.1037/adb0000862 35737551

[36] Kinley I Amlung M Becker S. Pathologies of precision: A Bayesian account of goals, habits, and episodic foresight in addiction. *Brain Cogn.* (2022) 158:105843. 10.1016/j.bandc.2022.105843 35066361

[37] Luhmann CC Ishida K Hajcak G. Intolerance of uncertainty and decisions about delayed, probabilistic rewards. *Behavior Ther.* (2011) 42:378–86. 10.1016/j.beth.2010.09.002 21658521

[38] Szpunar KK. Episodic future thought: An emerging concept. *Perspect Psychol Sci.* (2010) 5:142–62. 10.1177/1745691610362350 26162121

[39] Hill PF Emery LJ. Episodic future thought: Contributions from working memory. *Conscious Cogn.* (2013) 22:677–83. 10.1016/j.concog.2013.04.002 23681207

[40] Zavagnin M De Beni R Borella E Carretti B. Episodic future thinking: the role of working memory and inhibition on age-related differences. *Aging Clin Exp Res.* (2016) 28:109–19. 10.1007/s40520-015-0368-6 25963665

[41] Snider SE Deshpande HU Lisinski JM Koffarnus MN LaConte SM Bickel WK. Working memory training improves alcohol users’ episodic future thinking: A rate-dependent analysis. *Biol Psychiatry Cogn Neurosci Neuroimaging.* (2018) 3:160–7. 10.1016/j.bpsc.2017.11.002 29529411 PMC5851289

[42] Garzón B Kurth-Nelson Z Bäckman L Nyberg L Guitart-Masip M. Investigating associations of delay discounting with brain structure, working memory, and episodic memory. *Cereb Cortex.* (2022):bhac164. 1–10. 10.1093/cercor/bhac164 35488441 PMC9977379

[43] Bickel WK Yi R Landes RD Hill PF Baxter C. Remember the future: working memory training decreases delay discounting among stimulant addicts. *Biol. Psychiatry.* (2011) 69:260–5. 10.1016/j.biopsych.2010.08.017 20965498 PMC3015021

[44] Zhao X Wang Y Maes JH. The effect of working memory capacity and training on intertemporal decision making in children from low-socioeconomic-status families. *J Exp Child Psychol.* (2022) 216:105347. 10.1016/j.jecp.2021.105347 34971975

[45] Azfar O. Rationalizing hyperbolic discounting. *J Econ Behav Organ.* (1999) 38:245–52. 10.1016/S0167-2681(99)00009-8

[46] Snider SE LaConte SM Bickel WK. Episodic future thinking: expansion of the temporal window in individuals with alcohol dependence. *Alcohol Clin Exp Res.* (2016) 40:1558–66. 10.1111/acer.13112 27246691 PMC5497459

[47] Stein JS Wilson AG Koffarnus MN Daniel TO Epstein LH Bickel WK. Unstuck in time: episodic future thinking reduces delay discounting and cigarette smoking. *Psychopharmacology.* (2016) 233:3771–8. 10.1007/s00213-016-4410-y 27553824 PMC9812225

[48] Hayman M. Two minute clinical test for measurement of intellectual impairment in psychiatric disorders. *Arch Neurol Psychiatry.* (1942) 47:454–64. 10.1001/archneurpsyc.1942.02290030112010 619839

[49] Klingberg T. Training and plasticity of working memory. *Trends Cogn Sci.* (2010) 14:317–24. 10.1016/j.tics.2010.05.002 20630350

[50] Myerson J Green L Warusawitharana M. Area under the curve as a measure of discounting. *J Exp Anal Behav.* (2001) 76:235–43. 10.1901/jeab.2001.76-235 11599641 PMC1284836

[51] Brown SW. Attentional resources in timing: Interference effects in concurrent temporal and nontemporal working memory tasks. *Percept Psychophys.* (1997) 59:1118–40. 10.3758/BF03205526 9360484

[52] Kluger BM Krupp LB Enoka RM. Fatigue and fatigability in neurologic illnesses: proposal for a unified taxonomy. *Neurology.* (2013) 80:409–16. 10.1212/WNL.0b013e31827f07be 23339207 PMC3589241

[53] Szpunar KK Spreng RN Schacter DL. A taxonomy of prospection: Introducing an organizational framework for future-oriented cognition. *Proc Natl Acad Sci USA.* (2014) 111:18414–21. 10.1073/pnas.1417144111 25416592 PMC4284580

[54] Addis DR Wong AT Schacter DL. Remembering the past and imagining the future: common and distinct neural substrates during event construction and elaboration. *Neuropsychologia.* (2007) 45:1363–77. 10.1016/j.neuropsychologia.2006.10.016 17126370 PMC1894691

[55] Boyer P. Evolutionary economics of mental time travel? *Trends Cogn Sci.* (2008) 12:219–24. 10.1016/j.tics.2008.03.003 18468941

[56] Buckner RL Carroll DC. Self-projection and the brain. *Trends Cogn Sci.* (2007) 11:49–57. 10.1016/j.tics.2006.11.004 17188554

[57] Na S Chung D Hula A Perl O Jung J Heflin M Humans use forward thinking to exploit social controllability. *Elife.* (2021) 10:e64983. 10.7554/eLife.64983 34711304 PMC8555988

[58] Schacter DL Addis DR. The cognitive neuroscience of constructive memory: remembering the past and imagining the future. *Philos Trans R Soc B Biol Sci.* (2007) 362:773–86. 10.1098/rstb.2007.2087 17395575 PMC2429996

[59] Szpunar KK Watson JM McDermott KB. Neural substrates of envisioning the future. *Proc Natl Acad Sci USA.* (2007) 104:642–7. 10.1073/pnas.0610082104 17202254 PMC1761910

[60] Shah A Kraemer KR Won CR Black S Hasenbein W. Developing digital intervention games for mental disorders: A review. *Games Health J.* (2018) 7:213–24. 10.1089/g4h.2017.0150 30106642

[61] Lempert KM Phelps EA. The malleability of intertemporal choice. *Trends Cogn Sci.* (2016) 20:64–74. 10.1016/j.tics.2015.09.005 26483153 PMC4698025

[62] Ariely D Loewenstein G. When does duration matter in judgment and decision making? *J Exp Psychol Gen.* (2000) 129:508. 10.1037/0096-3445.129.4.508 11142865

[63] Ebert JE Prelec D. The fragility of time: Time-insensitivity and valuation of the near and far future. *Manag Sci.* (2007) 53:1423–38. 10.1287/mnsc.1060.0671 19642375

[64] Loewenstein GF. Frames of mind in intertemporal choice. *Manag Sci.* (1988) 34:200–14. 10.1287/mnsc.34.2.200 19642375

[65] Faralla V Novarese M Di Giovinazzo V. Replication: Framing effects in intertemporal choice with children. *J Econ Psychol.* (2021) 82:102345. 10.1016/j.joep.2020.102345

[66] Reeck C Figner B Weber EU Steffener J Krosch AR Wager TD Framing the future first: Medial temporal lobe activation discriminates delay and acceleration framing in intertemporal choice. *J Neurosci Psychol Econ.* (2021) 14:71. 10.1037/npe0000122

[67] DeSteno D Li Y Dickens L Lerner JS. Gratitude: A tool for reducing economic impatience. *Psychol Sci.* (2014) 25:1262–7. 10.1177/0956797614529979 24760144

[68] Guan S Cheng L Fan Y Li X. Myopic decisions under negative emotions correlate with altered time perception. *Front Psychol.* (2015) 6:468. 10.3389/fpsyg.2015.00468 25941508 PMC4400848

[69] Smallwood J Brown KS Tipper C Giesbrecht B Franklin MS Mrazek MD Pupillometric evidence for the decoupling of attention from perceptual input during offline thought. *PLoS One.* (2011) 6:e18298. 10.1371/journal.pone.0018298 21464969 PMC3064669

[70] Smallwood J Ruby FJ Singer T. Letting go of the present: mind-wandering is associated with reduced delay discounting. *Conscious Cogn.* (2013) 22:1–7. 10.1016/j.concog.2012.10.007 23178292

[71] Brooks SK Webster RK Smith LE Woodland L Wessely S Greenberg N The psychological impact of quarantine and how to reduce it: rapid review of the evidence. *Lancet.* (2020) 395:912–20. 10.1016/S0140-6736(20)30460-8 32112714 PMC7158942

[72] Pfefferbaum B North CS. Mental health and the Covid-19 pandemic. *N Engl J Med.* (2020) 383:510–2. 10.1056/NEJMp2008017 32283003

[73] Clay JM Parker MO. Alcohol use and misuse during the COVID-19 pandemic: a potential public health crisis? *Lancet Public Health.* (2020) 5:e259. 10.1016/S2468-2667(20)30088-8 PMC7195126 32277874

[74] Johnson SL Porter P Modavi K Dev A Pearlstein J Timpano KR. Emotion-related impulsivity predicts increased anxiety and depression during the COVID-19 pandemic. *J Affect Disord.* (2022) 301:289–99. 10.1016/j.jad.2022.01.037 35026359 PMC8747782

[75] Park J Lee S Sul S Chung D. Depression symptoms mediate mismatch between perceived severity of the COVID-19 pandemic and preventive motives. *Front Psychol.* (2021) 12:650042. 10.3389/fpsyg.2021.650042 34366971 PMC8339883

[76] Gobet F Sala G. Cognitive training: A Field in Search of a Phenomenon. *Perspect Psychol Sci.* (2022) 1–17. 10.1177/17456916221091830 35939827 PMC9903001

[77] Davis C Patte K Curtis C Reid C. Immediate pleasures and future consequences. A neuropsychological study of binge eating and obesity. *Appetite.* (2010) 54:208–13. 10.1016/j.appet.2009.11.002 19896515

[78] Jarmolowicz DP Cherry JB Reed DD Bruce JM Crespi JM Lusk JL Robust relation between temporal discounting rates and body mass. *Appetite.* (2014) 78:63–7. 10.1016/j.appet.2014.02.013 24650831 PMC4220169

[79] Stoeckel LE Murdaugh DL Cox JE Cook EW III Weller RE. Greater impulsivity is associated with decreased brain activation in obese women during a delay discounting task. *Brain Imaging Behav.* (2013) 7:116–28. 10.1007/s11682-012-9201-4 22948956 PMC3561478

[80] Han R Takahashi T Miyazaki A Kadoya T Kato S Yokosawa K. Activity. in the left auditory cortex is associated with individual impulsivity in time discounting. New York: In *Proceedings of the 2015 37th Annual International Conference of the IEEE Engineering in Medicine and Biology Society (EMBC).* (2015). 10.1109/EMBC.2015.7319917 26737817

[81] Kirby KN Petry NM. Heroin and cocaine abusers have higher discount rates for delayed rewards than alcoholics or non-drug-using controls. *Addiction.* (2004) 99:461–71. 10.1111/j.1360-0443.2003.00669.x 15049746

[82] Power Y Goodyear B Crockford D. Neural correlates of pathological gamblers preference for immediate rewards during the Iowa gambling task: an fMRI study. *J Gambl Stud.* (2012) 28:623–36. 10.1007/s10899-011-9278-5 22037936

[83] Watts TW Duncan GJ Quan H. Revisiting the marshmallow test: A conceptual replication investigating links between early delay of gratification and later outcomes. *Psychol Sci.* (2018) 29:1159–77. 10.1177/0956797618761661 29799765 PMC6050075

